# Thirty years of blackouts: a case report of swallow syncope

**DOI:** 10.3402/jchimp.v3i1.20323

**Published:** 2013-04-17

**Authors:** Irene Lambiris, Ivan Mendoza, Marcelo Helguera, Jose Baez Escudero, Cesar Bonilla

**Affiliations:** Cleveland Clinic Florida, Weston, FL, USA

**Keywords:** AV block, deglutition syncope, vagotonic hypersensitivity, dual chamber pacemaker, tilt-table, electophysiologic testing

## Abstract

Deglutition syncope has been demonstrated in isolated case reports, the first being described over 50 years ago. It is thought to be caused by a hypersensitive vagotonic reflex in response to esophageal dilation after swallowing. It can cause syncope due to complete atrioventricular (AV) block and acute reduction of cardiac output. Although rare, its lethality is worthy of discussion, as early recognition can offer complete treatment with placement of a pacemaker. A 54-year-old man presented with 30 years of lightheadedness and syncope, followed by disorientation and tremors, after eating sandwiches or drinking carbonated beverages. He initially was evaluated by a neurologist. Work-up included cardiac 2D transthoracic echocardiogram, electroencephalogram, swallow stud, pulmonary function tests, electrocardiogram, and cardiac stress testing. All tests were within normal limits, and it was determined that he was suffering from convulsive syncope and deglutition syncope. Referral to the cardiac electrophysiology department with tilt-table testing accompanied by swallow evaluation was then recommended. The tests demonstrated marked vagal response resulting in sinus bradycardia with second-degree AV block and pauses up to 3.5 seconds. Patient experienced near syncope. A rate-responsive, dual-chamber Boston Scientific pacemaker with DDDR programming was implanted. Patient has remained asymptomatic at follow-up.

ST, a generally healthy 54-year-old man, presented to the outpatient department for episodes of syncope, near syncope, and lightheadedness after eating. These episodes began at age 24. Since then, he has had these events with variable frequency. Initially, they would occur 1–2 times per year. As time went on, the episodes increased to 1–2 times per week. He stated that these episodes would occur after eating sandwiches, hamburgers, or drinking carbonated beverages. Specifically, he would experience a ‘choking’ sensation, difficulty breathing, and then would lose consciousness immediately upon swallowing. He would remain unconscious for less than a minute. There would be an ‘aura’, prior to the event, described as a sensation of nausea, dizziness, sweating, weakness, and sensation of increased body temperature.

Upon awakening, he would feel dazed. He denied chest pain, palpitations, cough, or lower extremity edema. Witnesses to the events told him he would be staring blankly, become acutely pale, and then would convulse and pass out. He had no tongue biting, and bowel or bladder incontinence. He had no personal or family history of myocardial infarction, arrhythmias, sudden death, or seizures. However, he injured himself on two occasions. In 2002, he was hospitalized at another facility. The emergency department discharged him after advising him that he had been choking and needed to modify his eating habits. He did modify his diet; he avoided carbonated beverages and hamburgers, as he felt these types of foods precipitated his symptoms. This actually helped reduce the number of syncopal episodes, although he still experienced near syncope. The frequency of events and the amount of time he would be unconscious was getting increasingly longer as he got older. Due to these longer and more frequent syncopal episodes, he decided to get re-evaluated.

Initially examined by our neurology department, his physical examination, vital signs (including orthostatics), and routine laboratory tests were normal. Electrocardiography showed normal sinus rhythm, rate of 74 beats per minute (bpm), with normal PR, QRS, and QT intervals. Chest x-ray was normal.

Echocardiogram showed stage 1 diastolic dysfunction with a left ventricular ejection fraction of 55–60% and no other valvular abnormalities. A brain MRI test from another facility was normal. Exercise stress test using an accelerated Bruce protocol was normal with no documented arrhythmias and excellent exercise tolerance. A 24-hour electroencephalogram showed no seizure activity. Barium swallow study showed normal esophageal anatomy and function. Pulmonary function testing revealed mild obstruction and tilt-table testing was normal.

Electrophysiologic testing (in a fasting state) was performed. Continuous blood pressure and cardiac monitoring were recorded. A carotid massage did not evoke any abnormal rhythms. The tilt-table test did not reproduce any symptoms; blood pressure and heart rate remained stable. The patient was given sublingual nitroglycerin of 0.2 mg with no reproduction of symptoms or abnormal response of vital signs. Patient was then asked to ingest a cold 500 ml carbonated beverage. Upon ingesting the beverage with one large swallow, the event monitoring showed marked vagal response.

A sinus arrest of 3.5 seconds and second-degree Mobitz Type 1 atrioventricular (AV) block occurred immediately after the first swallow. Patient did describe symptoms of lightheadedness and near syncope at that time. The positive study confirmed swallow syncope as the etiology of the patient's symptoms. A dual-chamber (Boston Scientific Ingenio) pacemaker was implanted, in DDDR mode, rate 60–120 bpm. Rate drop feature (sudden bradycardic response) was activated. Subsequently, he has had no more syncopal episodes, although he continues to have the sensation of near syncope (nausea and lightheadedness) upon swallowing (Fig. 1).

**Fig. 1:**
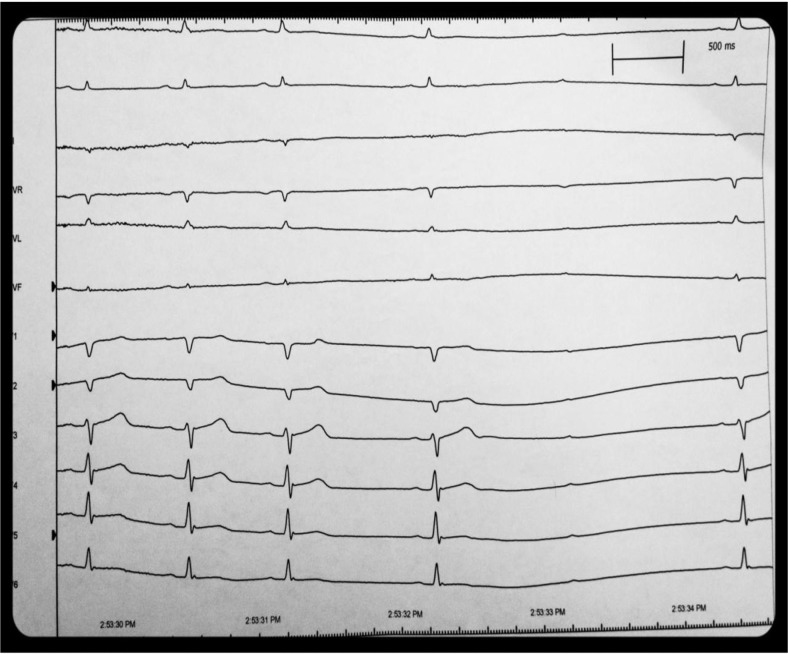
Cardiac monitoring following ingestion of 500 ml of a cold carbonated beverage. The patient had marked sinus bradycardia of 30 beats per minutes after the one sip. Following the bradycardia was a 3.5-second sinus pause with second-degree Mobitz Type 1 AV block. He complained of near syncope during the event.

## Discussion

The causes of syncope can be categorized into three groups; 48% non-cardiogenic (including reflex syncope and orthostatic hypotension), 18% cardiogenic, and 34% unknown. Of the non-cardiogenic, 5% can be attributed to situational syncope syndromes, including defecation, micturition, cough, and swallow. It is rare to have cardioinhibition from situational syncope, as we had in our patient ST. Esophageal disease, along with cardiac conditions, including myocardial infarction, rheumatic myocarditis, valvulopathy, aortic calcification, and AV nodal blocking agents have all been known to aggravate cardioinhibition. Interestingly enough, our patient lacked organic disease of the heart or esophagus.

The postulated mechanism for swallow syncope involves aberrant conduction of the afferent and efferent impulses of the vagus nerve. We can suspect that lower esophageal mechanoreceptors play a role, since symptoms were alleviated in our patient with restriction of certain beverages and foods that exacerbate esophageal dilation (1, 2). The hypersensitive gastrointestinal cardiac vasovagal reflexes which activate a variety of brady-tachyarrythmias by sympathetic inhibition produce a sufficient enough drop in cardiac output to cause syncope.

The vast majority of patients with syncope describe an aura, different from the aura described in epilepsy. The most common sensations are nausea, dizziness or lightheadedness, visual impairment, or ‘weakness’. The seizure-like activity experienced by our patient, ST, was defined by our neurologist as convulsive syncope. It is very common for syncopal episodes to be accompanied by loss of muscle tone and a lack of tonic or clonic activity. When tonic or clonic movements have been seen, the term ‘convulsive syncope’ has been used.

Although medications were not used in this case, atropine and other inhibitors of vagal conduction have had some success in reduction of syncope. A negative consequence is intolerable side effects, which limit widespread use. In our patient, it seemed that as he progressed in age, so did the severity of the syncopal events; thus, earlier intervention may have prevented needless morbidity and mortality. Intervention should include earlier referral to the appropriate specialists; in this case, the cardiac electophysiologist that can prescribe event monitoring and perform tilt-table testing with temporary pacing pre- and post-AV block conduction. In the case of ST, his tilt testing was classified as a Type 2B positive response by the Vasovagal Syncope International Study (VASIS). This is defined as cardioinhibition with asystole >3 seconds.

In our case of ST, this was not a simple neurocardiogenic syncope/situational syncope, wherein the controversy for implantation of PPM lies. In this instance, the cardioinbition was distinct from the vasodepressive affect from swallowing, thus increasing risk of potential fatal complications. ST required definitive treatment to prevent cardiac death. The 2012 Heart Rhythm Society (HRS)/American College of Cardiology (ACCF) guidelines for device placement in patients with neurocardiogenic syncope and asystole for >3 seconds are a Class 2a Level of Evidence C indication. This means that the weight of evidence for implanting a dual-chamber pacemaker is in favor of efficacy, with data derived from a consensus opinion of experts, case studies, or standard of care. Efficacy is enhanced by early intervention thus necessitating a higher sensitivity for this syndrome. A correct diagnosis will ensure appropriate tests and referrals to appropriate specialists, thus reducing needless expenditures.
